# Simulation and Thermodynamic Evaluation of Woody Biomass Waste Torrefaction

**DOI:** 10.1021/acsomega.4c08299

**Published:** 2025-01-21

**Authors:** Thiago da Silva Gonzales, Simone Monteiro, Giulia Cruz Lamas, Pedro P. O. Rodrigues, Mario B. B. Siqueira, Luis Alberto Follegatti-Romero, Edgar A. Silveira

**Affiliations:** † Mechanical Sciences Graduate Program, Laboratory of Energy and Environment, University of Brasília, Federal District Brasilia 70910-900, Brazil; ‡ Laboratory of Separation and Purification Engineering (LaSPE), Department of Chemical Engineering (PQI), Polytechnic School (EP), University of São Paulo (USP), São Paulo 05508-070, São Paulo, Brazil

## Abstract

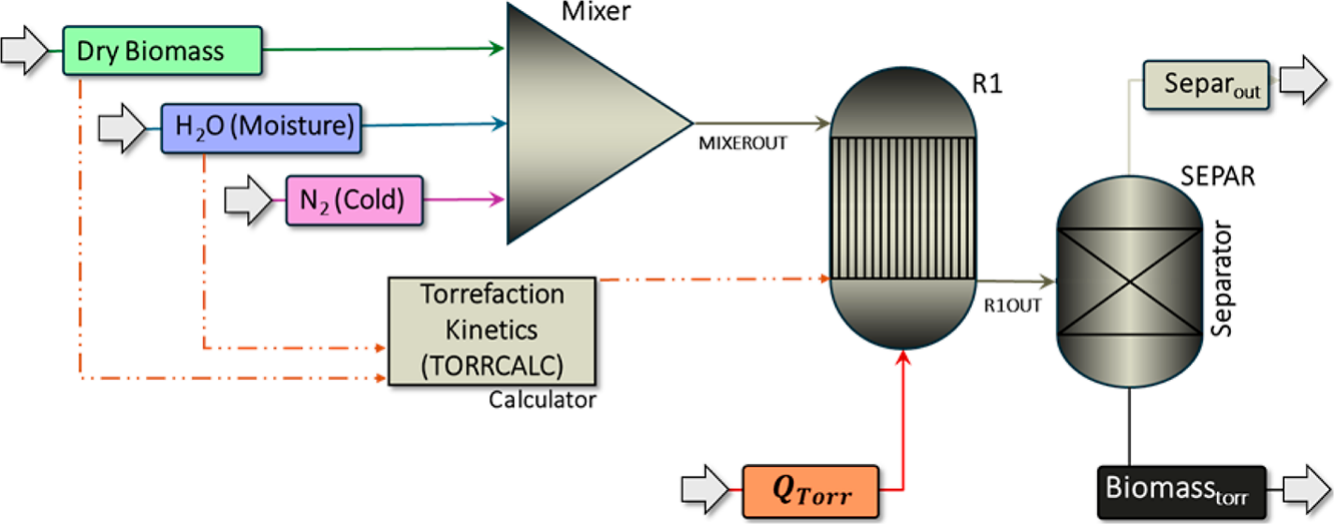

Torrefaction is a thermochemical pretreatment that enhances biomass properties, improving energy density, decomposition resistance, and hydrophobicity, making it a viable alternative as biofuel. This study performed a thermodynamic assessment of the torrefaction process for urban forest waste, integrating experimental data with two-step reaction kinetic modeling to evaluate the torrefaction product yields and properties using Aspen Plus software. The process was modeled with a yield reactor, employing the Peng–Robinson equation to describe vapor-phase behavior and empirical correlations to predict solid-phase properties. Simulations were validated against experimental data for temperatures between 225 and 275 °C, achieving an absolute deviation of less than 5%. Energy consumption ranged from 368 kJ·h^–1^ for light torrefaction to 1853 kJ·h^–1^ for severe torrefaction. Process irreversibility varied from 326 kJ·h^–1^ (3% exergy destruction) in light torrefaction to 3993 kJ·h^–1^ (16% exergy destruction) in severe torrefaction. The research provides a robust model for torrefaction scale-up that is adaptable to diverse biomass feedstocks and process conditions, highlighting its potential for optimizing energy use and improving sustainability in biomass utilization.

## 1 Introduction

The growing population and rising energy demand have intensified the search for sustainable solutions to minimize environmental impacts and enhance waste management. High fossil fuel prices and mounting environmental concerns have further driven this pursuit. While economic and technological development has underscored the importance of energy, it has also contributed to environmental degradation, emphasizing the need to diversify the energy matrix.^1,2^

Biomass is currently the fourth largest energy source after coal, oil, and natural gas, making it one of the most significant forms of renewable energy available worldwide.^3^ Due to its abundance and renewable nature, biomass is a strong candidate as an alternative to coal.^4^ In the future, solid biofuels derived from biomass are expected to become a major energy source.^5^ However, advanced technologies are required to convert and upgrade biomass into a coal-like biofuel to utilize it effectively. Raw biomass presents several challenges, including low energy density, high oxygen and moisture content, hygroscopic nature, and highly variable composition.^6,7^ Specifically, the lignocellulosic biomass presents structural heterogeneity, leading to nonuniform physical properties.^8^ These characteristics complicate its handling, transportation, storage, and conversion processes.^9,10^

Literature indicates that torrefaction is a promising and essential pretreatment for lignocellulosic biomass.^9,11^ Torrefaction is a thermochemical process typically conducted in an inert or partially oxidative atmosphere at temperatures between 200 and 300 °C for 20–60 min.^4,12^ Recent studies have also explored torrefaction in the transition zone to pyrolysis (350 °C)^13^ and under modified atmospheres, such as CO_2_^14,15^ and flue gas,^16^ to enhance biomass properties and energy efficiency. The resulting biocoal acquires coal-like properties, optimized for power and heat generation, with enhanced energy density, hydrophobicity, decomposition resistance, and improved storage performance.^9,17^

Torrefaction is an encouraging technology with a global Technology Readiness Level ranging from 6 to 8, reflecting progress from pilot-scale operations to limited commercial applications.^18^ This proves its scalability and adaptability for a sustainable biomass deployment. However, further research is essential to overcome economic barriers and enable broader deployment, particularly in underdeveloped countries. The literature highlights key areas for improvement, including advancements in kinetics, reactor design, fuel flexibility, process control, and scale-up modeling.^9,12^

The torrefaction process has been extensively investigated through experimental and numerical methods, focusing on the reaction kinetics and process simulation. Studies on reaction kinetics for biomass degradation and their reaction mechanisms usually focus on thermogravimetric data to predict the solid yield. On the other hand, the process simulation considers the entire reactor system, energy expenditures, and the potential for upscaling.

Numerous studies have demonstrated that Aspen Plus software is a reliable tool for modeling and predicting torrefaction outcomes.^19^ Previous studies primarily focused on mass and energy yields, the quantification of the Higher Heating Value (HHV), the characterization of torrefied biomass, and the composition of volatile compounds under specific conditions. Various feedstocks were explored, including pine wood chips, corn residues, forest residues, coffee husks and grounds, *Pinus radiata*, and *Eucalyptus globulus*. Onsree et al.^19^ studied the yield and energy requirements for torrefaction of corn residue pellets, while Mukherjee et al.^20^ studied the effect of torrefaction parameters on product and byproduct composition of Spent Coffee Grounds and Coffee Husk. On the modeling side, it is worth mentioning the work of Bach et al.,^21,22^ which presented a complete biomass torrefaction model applied to analyze the process efficiencies, and Manouchehrinejad and Mani,^23^ who simulated the integration of biomass torrefaction and pelletization plant.

While these studies generally focus on relevant biomass properties and energy consumption, they often overlook the assessment of energy quality (exergy) related to chemical transformations of biomass. In this context, Arteaga-Pérez^24^ evaluated integrated drying torrefaction using energy and exergy criteria. Their work highlights the importance of an integrated first- and second-law thermodynamic analysis of the torrefaction process.

Exergy analysis allows improvements in energy efficiency by evaluating process performance and identifying opportunities for enhancements in high-energy-consuming equipment. This approach assesses the efficiency of individual system components and identifies potential solutions for optimizing overall system performance.^25^ In contrast to energy analysis, it considers both the quantity and quality of energy, making it essential for sustainability and Life Cycle Assessment (LCA) studies.^26,27^

The originality of this study lies in (i) developing a detailed torrefaction plant model in Aspen Plus, enabling a comprehensive analysis of process outcomes; (ii) integrating experimental torrefaction data, including raw material composition and two-step reaction kinetics, for model validation; (iii) performing a thermodynamic analysis of the torrefaction process, evaluating energy and exergy performance in a continuous plant designed to valorize urban forest waste (UFW); and (iv) conducting a thorough evaluation of torrefaction yields, energy requirements, and irreversibilities across varying temperatures, residence times, and moisture levels, using a central composite design to cover the full range of light, mild, and severe torrefaction. The results provide critical insights for future economic and environmental analyses, such as LCA, while paving the way for advanced exergoeconomic and exergoenvironmental studies. This research offers a robust and reliable model to serve as a tool for the complete assessment of torrefaction scale-up, which can be adapted to various biomass feedstocks and process conditions.

## 2 Methodology

### 2.1 Biomass Feedstock Modeling

Aspen Plus was chosen for its ability to model thermochemical processes such as biomass torrefaction, offering robust thermodynamic libraries, multiphase system tools, and seamless integration of experimental data. It offers a user-friendly interface, extensive thermodynamic libraries, robust multiphase system tools, and the seamless integration of experimental data with reaction kinetics. Additionally, it allows the incorporation of custom-built subroutines developed in Fortran or Excel^28^ for accurate simulations.

The primary challenge in thermochemical modeling of biomass conversion is accurately representing biomass with variable chemical and elemental compositions.^29^ Different solutions are available to estimate biomass thermochemical properties without considering the full complexity of its chemical composition. In this study, biomass and biocoal were treated as nonconventional solids without a specific formula, characterized by proximate, ultimate, and sulfur compositions.^30^ The proximate analysis gives the solid composition in terms of moisture, fixed carbon, volatile matter, and ash, denoted as PROXIMAL in Aspen Plus. The ultimate analysis gives the solid composition in terms of carbon, hydrogen, nitrogen, chlorine, sulfur, oxygen, and ash, denoted as ULTANAL. Additionally, the sulfur compositions give the weight fractions of sulfur divided into pyritic, sulfate, and organic sulfur, known as SULFANAL.^30,31^ These compositions are essential for accurate modeling, reaction understanding, and mass balance calculations.

For greater accuracy in calculating heating values for organic fuels like coal and biomass, it is recommended to use mineral matter-free mass fractions^32^ (eq 1). The mineral matter content is calculated using the modified Parr formula (eq 2), which considers both sulfatic and organic sulfur. When information on the different forms of sulfur is available, the modified Parr formula offers a more precise estimate of the percentage of inorganic material present.^30^
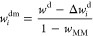
1
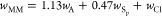
2where *w* corresponds to the mass fraction and the subscripts MM, A, S_p_, and Cl correspond to mineral matter, ash, pyritic sulfur, and chlorine. The superscripts d and dm corresponds to dry and dry and mineral and matter-free. Δ*w*_*i*_^d^ represents the correction factor for other losses. For carbon and hydrogen loss in the water, the constitution of clays was calculated according to eqs 3 and 4. Oxygen and organic sulfur were calculated by eqs 5 and 6, respectively.^30^
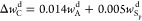
3
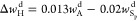
4
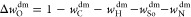
5
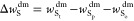
6

The properties calculated for nonconventional solids were density and enthalpy. The density was calculated by using the DCOALIGT model, which is based on the Institute of Gas Technology (IGT) correlation. This model provided the density of coal on a dry basis and was used to calculate the density of nonconventional components (biomass, coal, and ashes). The model requires ULTANAL and uses ultimate and sulfur compositions.^30,33^Equations 7–9 are those of the model based on IGT equations.^30^ The coefficients *a*_*i*_ used in these formulas can be found in Table S1 (Parameter name: DENIGT).
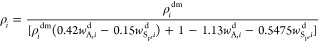
7
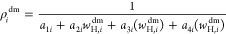
8
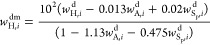
9

The general coal model (HCOALGEN) for calculating enthalpy includes various experimental data to estimate the heat of combustion, heat capacity, and heat of formation of nonconventional components.^30,33^ The combustion of coal in the HCOALGEN model uses a gross calorific value. A deduction for the latent heat of water vaporization is necessary to calculate the net calorific value. The heat of combustion values was converted to a dry, mineral-matter-containing basis, with a correction for the heat of combustion of pyrite (eq 10).^30^
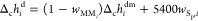
10

The constants in the HCOALGEN correlations are bias corrections derived from the IGT study.^30^ Numerous equations have been developed to estimate the heating value of organic fuels, such as coal, relying on the fuel’s elemental composition.^34^ The Aspen Plus software employs a variety of relevant literature correlations (Boie Correlation, Dulong Correlation, Grummel and Davis Correlation, Mott and Spooner Correlation, IGT Correlation, and Revised IGT Correlation) developed for coal or other organic fuels. For this study, the Boie correlation (eq 11) was chosen to estimate the heating value.^30,35^ The coefficients (*a*_*i*_) used in the simulations were obtained from internally integrated tabulated parameters, which can be consulted in Table S1 in the Supporting Information (Parameter name: BOIEC).
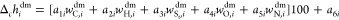
11

The energy balance of unit operations relies on the standard heat of formation of the participating substances. Thus, the standard heat of formation of nonconventional solids is calculated directly from the heating value of the substances. The heat-of-combustion-based correlation (eq 12) was chosen to estimate the heat of formation. This assumes that during combustion, all elements undergo complete oxidation except for sulfatic sulfur and ash, which are inert. The numerical coefficients are combinations of stoichiometric ratios and heat of formation for CO_2_, H_2_O, HCl, and NO_2_ at 298.15 K.^30^
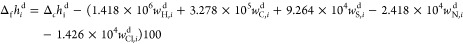
12

The Kirov Correlations for the heat capacity (eq 13) have been chosen to estimate the Heat Capacity (J·kg^–1^·K^–1^).^30,36^ The Kirov correlation considers coal a mixture of moisture, ash, fixed carbon, and primary and secondary volatile matter. Secondary volatile matter includes any volatile matter up to 10% on a dry, ash-free basis; the remaining volatile matter is considered primary.^30^ The coefficients can be consulted in Table S2 in the Supporting Information.
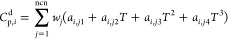
13

Equations 1–13 represent the built-in calculations within the Aspen Plus software through the nonconventional HCOALGEN and DCOALIGT models. Their inclusion in this study clarifies the methodology and ensures transparency in the simulation process. Primarily designed for coal, it successfully simulated torrefaction, delivering reliable and accurate results.^19,20,23^ Used to simulate the pretreatment of different lignocellulosic feedstocks, the software can model thermochemical conversion processes, including feedstock decomposition and the separation of condensable/noncondensable gases and solids/gases.^28^

The chosen feedstock was based on the literature on woody residues. Silveira et al.^17^ experimentally investigated a blend of UFW comprising six tree species, conducting torrefaction at temperatures between 225 and 275 °C, with a controlled heating rate of 7 °C·h^–1^ and residence times ranging from 20 to 60 min in an inert atmosphere. The proximate, ultimate, and calorific properties used as input data for the modeling feedstock are available in the Supporting Information (Table S3).

### 2.2 Torrefaction Plant Modeling

**Figure 1:**
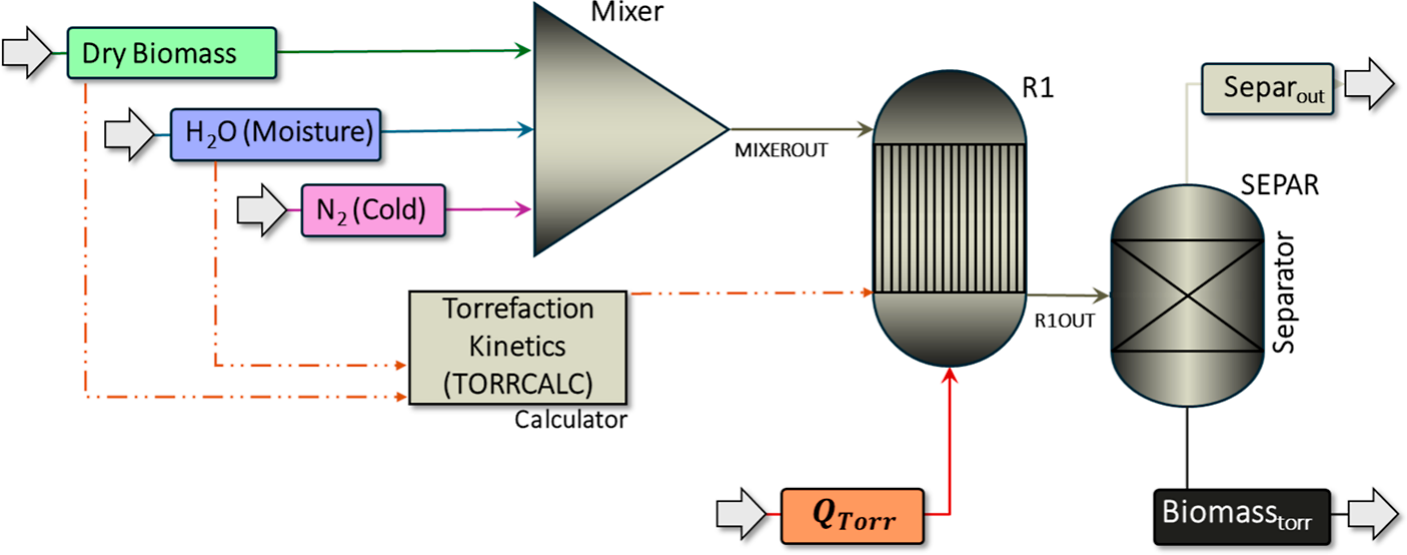
Torrefaction process in Aspen Plus.

The torrefaction process was simulated using Aspen Plus according to the proposed flowsheet (Figure 1). Different blocks were used for torrefaction simulations since no predefined reactors in Aspen Plus can adequately model this complex process.^22,28^ Since kinetic modeling offers greater accuracy than equilibrium modeling,^37^ reaction kinetics from the previous study^17^ was integrated with Aspen Plus by the CALCULATOR block. Silveira et al.^17^ employed a kinetic model based on two-step consecutive reactions^38,39^ (see Figure S1) to predict the torrefaction yield of UFW between 225 and 275 °C and residence times of 20–60 min in an inert atmosphere. This model, widely applied to various biomasses, effectively describes torrefaction kinetics under varied conditions.^38,39^ A comprehensive list of all evolved reactions and kinetic parameters used in this study can be found in the Supporting Information (Table S4).

The following considerations were assumed: (i) isothermal and at a steady-state process; (ii) the particle size effect and intraparticle heat and mass transfer were not considered; (iii) the Peng–Robinson equation of state with the Boston–Mathias alpha function described vapor–liquid equilibria; (iv) the biomass and biocoal produced were considered nonconventional components; (v) nitrogen was added to simulate an inert environment during torrefaction; and (vi) the reference state for the Exergy calculations was defined as *T*_0_ = 25 °C e *P*_0_ = 1.0 atm.

In Figure 1, the raw biomass was represented by the mixture of two streams: “Dry Biomass” (in green) and “H_2_O (Moisture)” (in blue). The “Dry Biomass” stream, based on a flow rate of 1 kg·h^–1^ (*T* = 25 °C; *P* = 1.0 atm), is characterized by its proximate and elemental composition. The “H_2_O (Moisture)” stream accounts for the moisture content of the biomass, ranging from 0 to 30%, with corresponding flow rates between 0.0 and 0.43 kg·h^–1^ (*T* = 25 °C; *P* = 1.0 atm). Additionally, the “N_2_ (Cold)” stream (in purple) represents the nitrogen (N_2_) input, with a flow rate of 1.2 mL·min^–1^ (*T* = 25 °C; *P* = 1.0 atm) of nitrogen for every 100 mg of material during torrefaction.^40^ All input streams were mixed in a Block Mixer (*P* = 1.0 atm).

The torrefaction process itself was simulated by using three blocks. The yield reactor (R1), RYield, operating at *T* = 225–275 °C and *P* = 1.0 atm, considered the decomposition of dried biomass following a two-step kinetic (Figure S1 in SM),^17,41^ with the aid of a TORRCALC block. The PR–BM property model (Peng–Robinson equation of state with Boston–Mathias modification) was used to estimate the properties of conventional components available in liquid and gas phases. The Separator block (SEPAR) separates the volatile material into the beige stream (Separ_out_) from the torrefied solid in the black stream (Biomass_torr_), operating at *T* = 225–275 °C and *P* = 1.0 atm. The produced volatiles (Table S5 in SM) were categorized as condensable (bio-oil), including acetic acid (CH_3_COOH), water (H_2_O), formic acid (HCOOH), methanol (CH_3_OH), lactic acid (CH_3_–CH(OH)–COOH), furfural (C_4_H_3_OCHO), hydroxyacetone (CH_3_COCH_2_OH),^42^ and the noncondensable (Torgas), including carbon dioxide (CO_2_) and carbon monoxide (CO).^42,43^ The red stream “*Q*_Torr_” represents the heat required for torrefaction calculated by the enthalpy difference between the R1OUT and the MIXEROUT streams.

Using the RYield block in combination with the CALCULATOR block was motivated by the feasibility of employing the kinetic equations (Table S4 in SM) to calculate the mass yields of the pseudocomponents (*A*, *B*, *C*, *V*_1_, and *V*_2_) considered in the two-step reaction model. This approach follows a methodology similar to that used by Puig-Gamero et al.,^44^ which used mass yield equations to simulate pyrolysis, employing an RYield reactor combined with a CALCULATOR block.

The studies by Arteaga-Pérez et al.,^24^ Mukherjee et al.,^20^ and Onsree et al.^19^ used similar configurations, though they employed two RYield reactors instead of the single one used in this work. In the works by Mukherjee and Onsree, the first reactor simulates the decomposition of biomass into volatiles and an intermediate solid, while the second reactor simulates the decomposition of the intermediate solid into volatiles and torrefied biomass. In Arteaga-Pérez’s study, the first reactor fractionates the wood into hemicelluloses, cellulose, and lignin, and the second reactor produces volatiles and torrefied solids. In all three studies, separators are used to isolate the volatile products from the torrefied solid products.

The present numerical assessment comprises two key analyses: (1) validation of the torrefaction plant with experimental data and (2) evaluation of the torrefaction process through modeling under all conditions within the validated range. Torrefaction was validated using experimental data obtained from torrefaction conducted at temperatures of 225–275 °C, with residence times ranging from 20 to 60 min and a heating rate of 7 °C·h^–1^ under an inert atmosphere.^17^ The kinetic reaction rates applied to model the thermodegradation were determined by evaluating biomass on a dry basis (0% moisture content).^17^

Next, the validated torrefaction plant was further evaluated for solid and energy yields, energy consumption, and irreversibilities, considering treatment temperatures of 225, 250, and 275 °C and residence times of 20, 40, and 60 min. In addition, energy requirements and irreversibilities were also analyzed concerning biomass moisture content levels of 5%, 17.5%, and 30%. A central composite face-centered design (CCD) was implemented, and the results were analyzed by using Stat-Ease-Design-Expert (version 23.1.4). These temperatures cover the three levels of torrefaction: light (200–240 °C), mild (240–260 °C), and severe (260–300 °C).^10^

#### 2.2.1 Exergetic Analysis

In contrast to energy analysis, exergy analysis offers a precise approach by considering both the quantity and the quality of energy. Exergy is closely linked to the sustainability and environmental impact of a process, making it an essential tool in LCA studies.^26^ For this analysis, the reference state for exergy calculations was defined at *T*_0_ = 25 °C and *P*_0_ = 1.0 atm.^45^ The physical exergy of biomass (both raw and torrefied) was not considered, as it is a solid.^46^ The exergy of a flow crossing the control volume boundary was calculated using eq 14, where factors temperature, pressure, and the reference state are denoted by subscript 0.^47,48^ The internal model of the EXERGYFL software calculated the physical exergy of the volatile flow in kJ·h^–1^.^30,45^
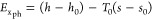
14In this case, *h* and *s* are the total enthalpies and entropies of the flow, respectively. The subscript 0 represents the dead state, i.e., *P* = 1 atm and *T* = 25 °C. Equation 15 determined the calculation of the chemical exergy of the biomass.^49^ The chemical exergy of the volatile mixture (in kJ·mol^–1^) was obtained by eq 16, requiring the standard chemical exergy value of chemical compound *i* (ex_ch,*i*_^0^) and *R* = 8.314 J K^–1^·mol^–1^.^50,51^
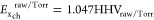
15
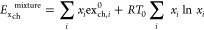
16Here, *x* represents the molar fraction. The first term in eq 16 represents the sum of each component’s chemical exergy contributions. The second term arises from the entropy generation associated with the mixture and depends on the concentration of each substance present.^47^ The standard chemical exergy value of a substance can be determined using eq 17, given the standard Gibbs free energy of formation (Δ*g*_r_^0^) and the standard chemical exergy of the constituent elements (ex_ch,element_^0^). The tabulated values for these properties, considering carbon, hydrogen, and oxygen, are ex_ch,C_^0^ = 410.26 kJ·mol^–1^,  = 236.1 kJ·mol^–1^, and  = 3.97 kJ·mol^–1^.^50,52^
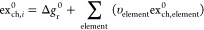
17Here, υ_element_ represents the stoichiometric coefficient. Table S6 lists the standard chemical exergy values for all nine components constituting the volatile material stream released during torrefaction and the standard chemical exergy of Nitrogen (N_2_) and Liquid Water. The standard chemical exergy values for nitrogen, water (in both liquid and vapor states), gaseous methanol, carbon dioxide, and carbon monoxide were obtained from tabulated data in the literature.^53^

The standard chemical exergy for the remaining elements was calculated using eq 17 and validated by comparing these values with those reported in the literature. For instance, the standard chemical exergy values for acetic acid, formic acid, lactic acid, and furfural were consistent with those from previous studies,^54,55^ confirming the appropriate use of the standard chemical exergy for hydroxyacetone. Additionally, the exergy of heat (in kJ) was calculated using eq 18 with *T*_Torr_ representing the torrefaction temperature.^50,51^
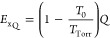
18

The exergetic balance^51^ (eq 19 in kJ) quantifies the input and output of the exergy, revealing the irreversibility of the process (*I*), denoted by the destroyed exergy.
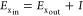
19

By evaluation of the destruction of exergy, which represents the useful part of energy, it is possible to accurately identify real losses in a process. This plays a crucial role in improving the efficiency and process optimization.

#### 2.2.2 Energy Performance of the Torrefied Product

The energy performance of the torrefied product was assessed by analyzing its HHV in MJ·kg^–1^ (eq 20),^1^ based on the elemental composition of biomass (C, H, S, O, and N in %). Additionally, the assessment included the enhancement factor (EF, dimensionless) (eq 21), energy yield (in %) (eq 22), and the energy-mass-*co*-benefit-index (EMCI, dimensionless) (eq 23), which represent the difference between energy yields (EYs) and mass yield (*Y*_TS_).
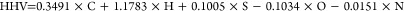
20
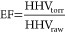
21
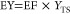
22
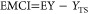
23

## 3 Results and Discussion

This section outlines the key analyses and interpretations derived from the study. The proposed simulation model was first validated through comprehensive comparisons with experimental data, confirming its accuracy and reliability. Following the validation, the effects of variables such as torrefaction temperature, residence time, and biomass moisture on the system’s responses were evaluated, providing a deeper understanding of the processes involved and their practical implications. These results are discussed in detail, highlighting their relevance and potential applications.

### 3.1 Validation of Torrefaction Process Simulation

**Figure 2:**
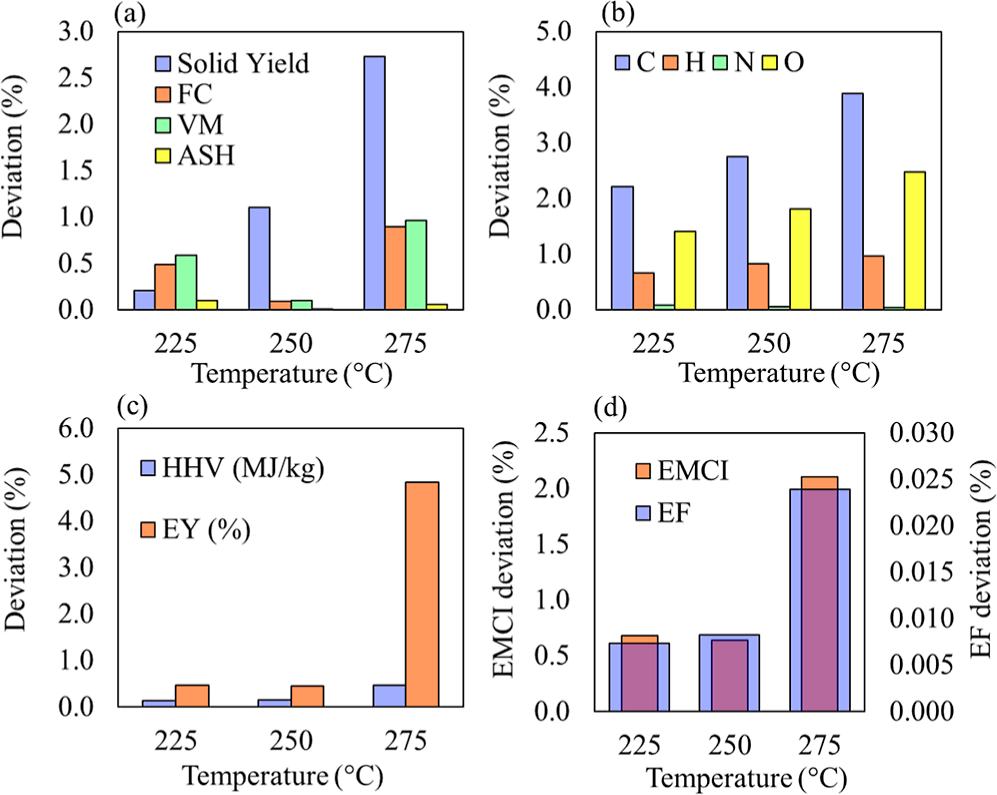
Absolute deviation (in %) for comparing simulation results with experimental data for (a) solid yield and proximate composition, (b) ultimate composition, (c) energy efficiency (EF) and energy-matter conversion index (EMCI), and (d) HHV and EY.

Table 1 presents the estimated (biocoal, bio-oil, and torgas) yields, proximate and ultimate compositions of biocoal, and energy indexes derived from the proposed model. These results were validated with experimental data from Silveira et al.^17^ (Figure 2).

**Table 1:** Simulation Results of Torrefaction Outcomes in Aspen Plus, Detailing the Product Yields, the Proximate and Ultimate Composition of Biocoal, Energy Performance Metrics, Exergy Flows, and Irreversibilities

	experimental data from Silveira et al.^17^				simulation		
	raw	225 °C	250 °C	275 °C	225 °C	250 °C	275 °C
Product Yields (%)							
biocoal (*Y*_ST_)	100.00	94.00	86.00	78.00	94.21	87.10	75.27
torgas					1.23	1.84	1.70
bio-oil					4.56	11.06	23.03
Proximate Compositions (%)^a^							
FC	17.90	19.88	24.31	30.20	20.37	24.40	31.10
VM	77.61	75.56	70.68	64.25	74.97	70.58	63.29
ash	4.49	4.56	5.01	5.55	4.66	5.02	5.61
Ultimate Compositions (%)^a^							
C	44.91	48.63	50.52	53.3	46.42	47.77	49.41
H	7.25	6.56	6.31	5.90	7.23	7.14	6.87
N	0.64	0.76	0.78	0.81	0.68	0.73	0.85
O^b^	42.71	39.49	37.38	34.44	40.9	39.19	36.91
H/C	1.92	1.61	1.49	1.32	1.86	1.78	1.66
O/C	0.71	0.61	0.56	0.49	0.66	0.62	0.56
HHV (MJ kg^–1^)							
biocoal	19.79	20.64	21.19	21.99	20.49	21.03	21.51
torgas					1.74	1.76	1.83
bio-oil					15.48	16.52	17.05
Energy Performance^a^							
EF		1.04	1.07	1.11	1.04	1.06	1.09
EY (%)		97.99	92.08	86.63	97.53	92.54	81.80
EMCI		3.99	6.08	8.63	3.31	5.44	6.53
Energy and Exergy							
*Q*_Torr_^c^					368	441	561
*E*_x__ch_^raw/Torr^^d^	20.73	21.61	22.19	23.02	21.45	22.02	22.52
*I*^c^					620	1581	3438

a Specifically for biocoal.

b O = 100–C–H–N–ash.

c MJ·h^–1^.

d (MJ·kg^–1^).

The simulation showed low deviations for all studied parameters, including solid yield, proximate and ultimate composition of a solid product, and energy indexes across the three analyzed temperatures. The largest deviations occurred at higher temperatures, which can be explained by the difficulty in conducting experiments under those conditions. The behavior of these responses aligned with the experimental results reported in the literature, indicating that the model is a reliable approach for describing the biomass and vapor–liquid equilibria.

The proposed torrefaction model tends to underestimate carbon content (C %) compared to experimental findings.^17^ Conversely, it overestimates H and O levels, increasing the discrepancy as the torrefaction process intensifies. These variations are directly linked to the fixed proportion of volatiles assumed in Bates’s work.^42^

The fixed proportion of volatiles, commonly used to model the torrefaction of various lignocellulosic biomasses in the literature,^56^ was originally derived from the investigation of Willow biomass. However, to achieve accurate predictions of volatiles, it is important to determine the specific volatile composition of the nine major components for each biomass. This limitation will be further explored in future research. Despite this, the model’s predictions for HHV, EF, EY, and EMCI correlated well with experimental data, with deviations remaining within acceptable limits as torrefaction severity increased.

Previous studies have addressed the simulation of the torrefaction process for various materials, including Norwegian birch,^22^ agricultural residues,^19^ and coffee husk with spent coffee grounds.^20^ The simulation of Norwegian birch showed solid yield values higher than experimental results, with differences up to 11.1% at 240 °C.^22^ In the case of agricultural residue pellets,^19^ deviations between numerical simulations and experimental data ranged from 5% to 12% at torrefaction temperatures of 260 and 300 °C. Moreover, Mukherjee et al.^20^ found a good correlation between the torrefaction of coffee husk and spent coffee grounds at 200 °C, but the model showed positive deviations at higher temperatures. As shown in Figure 2, the observed differences are consistent with those in the literature, confirming the reliability of the present model as a robust tool for evaluating the torrefaction process.

**Figure 3:**
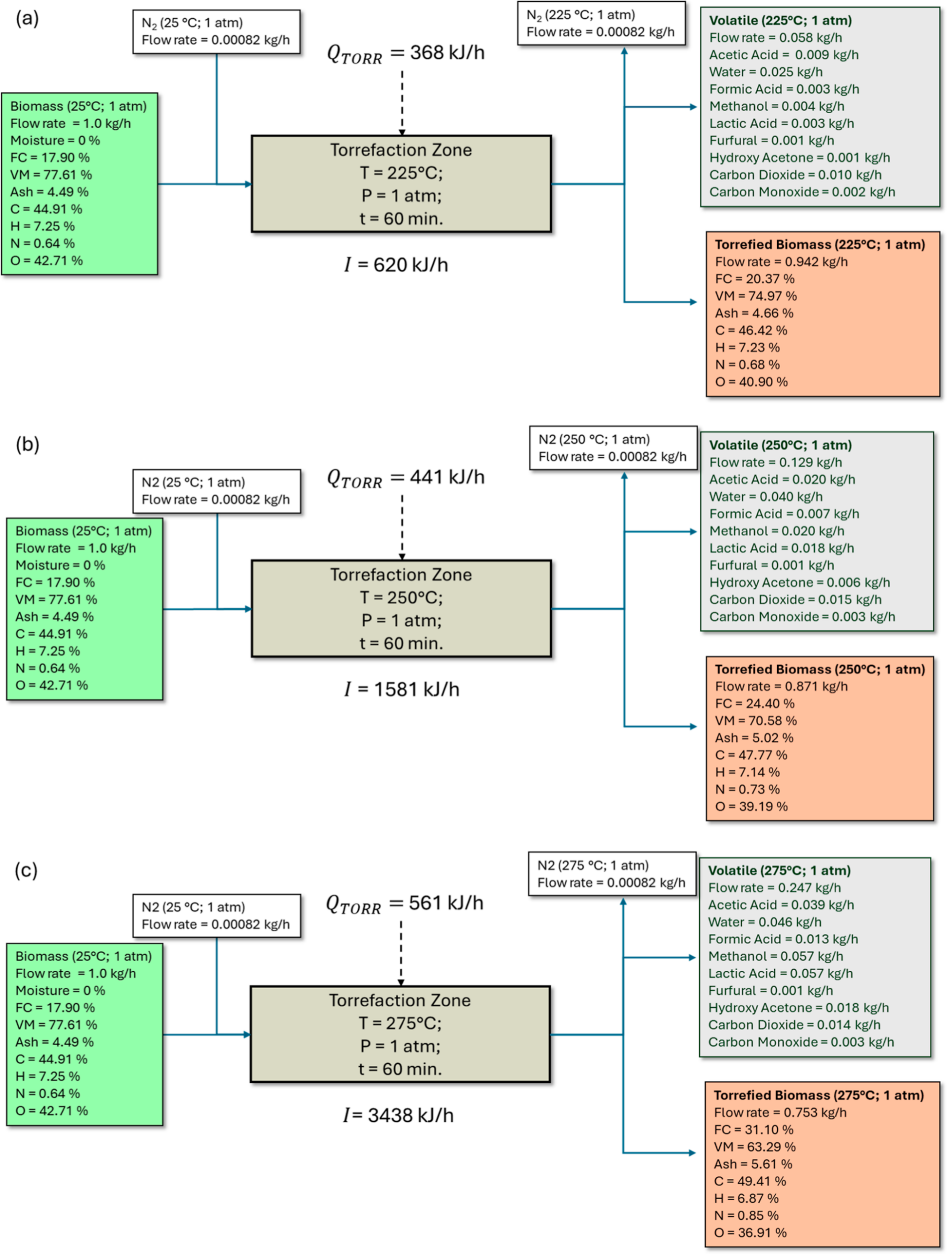
Mass, energy, and irreversibility flow for torrefaction: (a) light, (b) mild, and (c) severe.

Figure 3 shows the flowchart of the torrefaction process under the validation conditions, illustrating the mass and energy inflows and outflows and the process’s respective irreversibilities (see Table 1). Table S7 complements Table 1 by providing the stream data from Aspen Plus for torrefaction validation for light, mild, and severe torrefaction cases (60 min reaction time and *P* = 1 atm), detailing each component’s flow rates, compositions, and temperatures for each block in the simulation.

The heat (*Q*_Torr_) consumed for the torrefaction of 1 kg·h^–1^ biomass at temperatures of 225, 250, and 275 °C was 368, 441, and 561 kJ kg^–1^, respectively, over 60 min. These values are consistent with previous studies. For instance, Onsree et al.^19^ used 380 kJ·kg^–1^ for the torrefaction of pellets for 20 min at 260 °C, while Manouchehrinejad and Mani^23^ used approximately 537 kJ·kg^–1^ for torrefying wood chips with 10% moisture content for 30 min at 270 °C.

Bach et al.^21,22^ conducted torrefaction of Norwegian birch with 10% moisture, consuming between 415 and 532 kJ·kg^–1^ over 30 min at temperatures ranging from 240 to 270 °C. Although these values are similar in magnitude, the variations in the heat required for different biomasses are attributed to differences in biomass composition. These compositional differences directly impact the specific heat of the raw material and consequently influence heat consumption during torrefaction.

**Figure 4:**
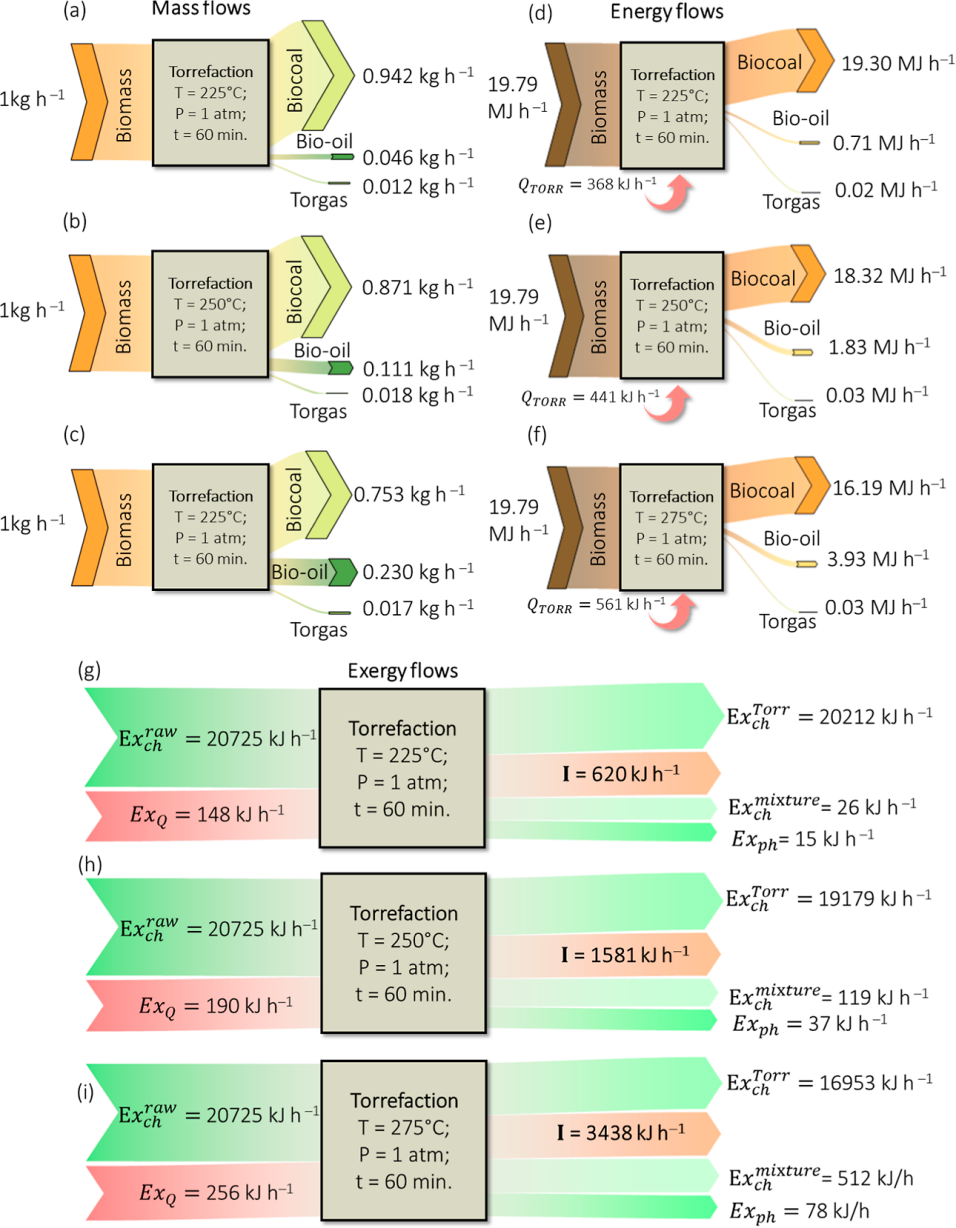
Mass (a–c), energy (d–f), and exergy (g–i) flow for light (225 °C), mild (250 °C), and severe (275 °C) torrefaction with 60 min residence time under inert conditions.

Figure 4 presents a Sankey diagram illustrating mass, energy, and exergy flows in the torrefaction process under validation conditions. The chart shows the amount of exergy transferred to each stream and highlights the process’s irreversibility. The following irreversibilities were observed for each pretreatment temperature: 620 kJ·h^–1^ (225 °C), 1581 kJ·h^–1^ (250 °C), and 3438 kJ·h^–1^ (275 °C) of unutilized energy. As the severity of torrefaction increases, the total exergy destruction (*I*) also rises. This phenomenon occurs due to significant irreversibilities associated with chemical reactions and heat transfer, which become more pronounced at higher temperatures. In torrefaction, the chemical exergy is significantly related to O/C and H/C ratios.^57^

When comparing Figure 4 and Table 1, the specific exergy of torrefied biomass is shown to increase with temperature. This result aligns with expectations, as severe torrefaction promotes higher energy densification. On the other hand, Figure 4 illustrates the exergy flow over time. It reveals that, although the exergy of the volatile flow (E_xph_ + E_x__ch_^mixture^) increases, the overall flow decreases with the increase, corresponding to an irreversibility increase. The destruction of the total exergy entering the system was 3% in the light treatment (225 °C), rising to approximately 8% in the mild process (250 °C) and reaching around 16% in the severe process (275 °C).

### 3.2 Assessment of Torrefaction by Simulation

#### 3.2.1 Temperature and Residence Time

**Figure 5:**
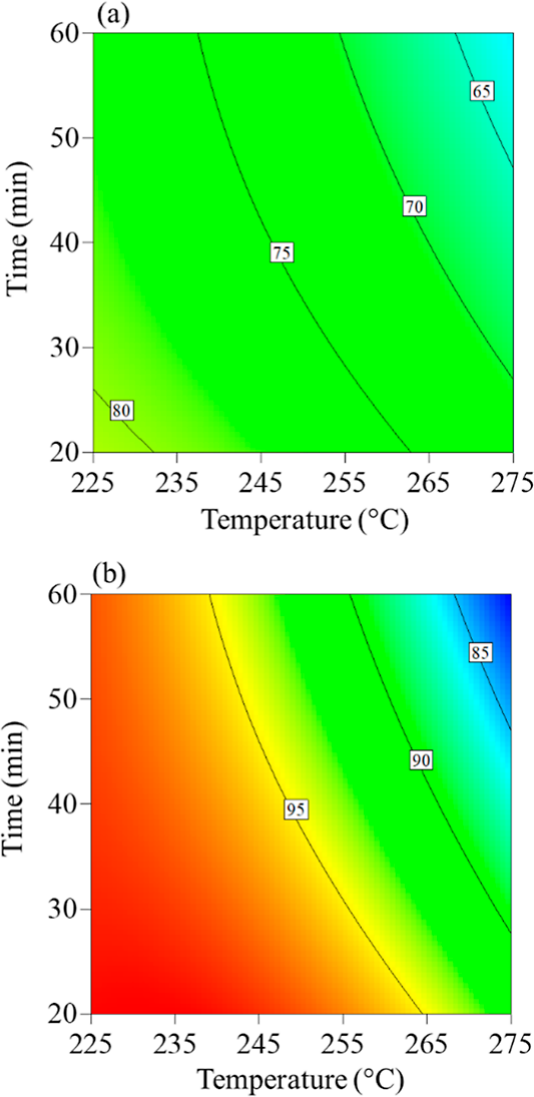
Contour results for solid yield (a) and energy yield (b) as a function of torrefaction time and temperature.

The simulation was conducted to analyze the influence of the treatment temperature and torrefaction time on solid and energy yields (Figure 5). The torrefaction process was varied between 225 and 275 °C, with residence times adjusted from 20 to 60 min.

First, the influence of residence time was evaluated. Figure 5a indicates that as the severity of torrefaction increases, solid yield values decrease, with temperature having a greater influence than residence time, at least over the range of temperature and time tested.^17,58^ During light torrefaction, low-molecular-weight volatiles are released. Hemicelluloses, the most reactive component, undergo thermal degradation, while cellulose and lignin are minimally affected.^8^ This results in a slight biomass weight loss and a modest increase in the energy density. During mild torrefaction, the breakdown of hemicelluloses and the release of volatiles become more pronounced.^59^ Hemicelluloses decrease significantly, and cellulose is also slightly impacted.^59^ Hemicelluloses are almost completely depleted in severe torrefaction, and cellulose is considerably degraded.^59^ Lignin remains relatively unaffected by the thermal process. This structural decomposition leads to a reduction in the solid yield (up to 22%) and an increase in the fuel’s energy density.^10,60^

The HHV of biomass increases with the severity of the torrefaction treatment, as does the EF, reflecting the expected process of energy densification.^61,62^ The EF is an index that measures improvements in the HHV of raw and torrefied biomass with EF values greater than 1.00, indicating energy densification after torrefaction.^63,64^ The EY (Figure 5b) represents the energy content retained in the torrefied product and is calculated by multiplying the solid yield by the EF.^65^ As the process temperature increases, volatiles are released due to water evaporation and the decomposition of low-molecular-weight compounds, primarily hemicelluloses. This decreases yield, leading to a linear reduction in EY despite an increase in the EF.^66,67^

#### 3.2.2 Residence Time and Raw Biomass Moisture Content

**Figure 6:**
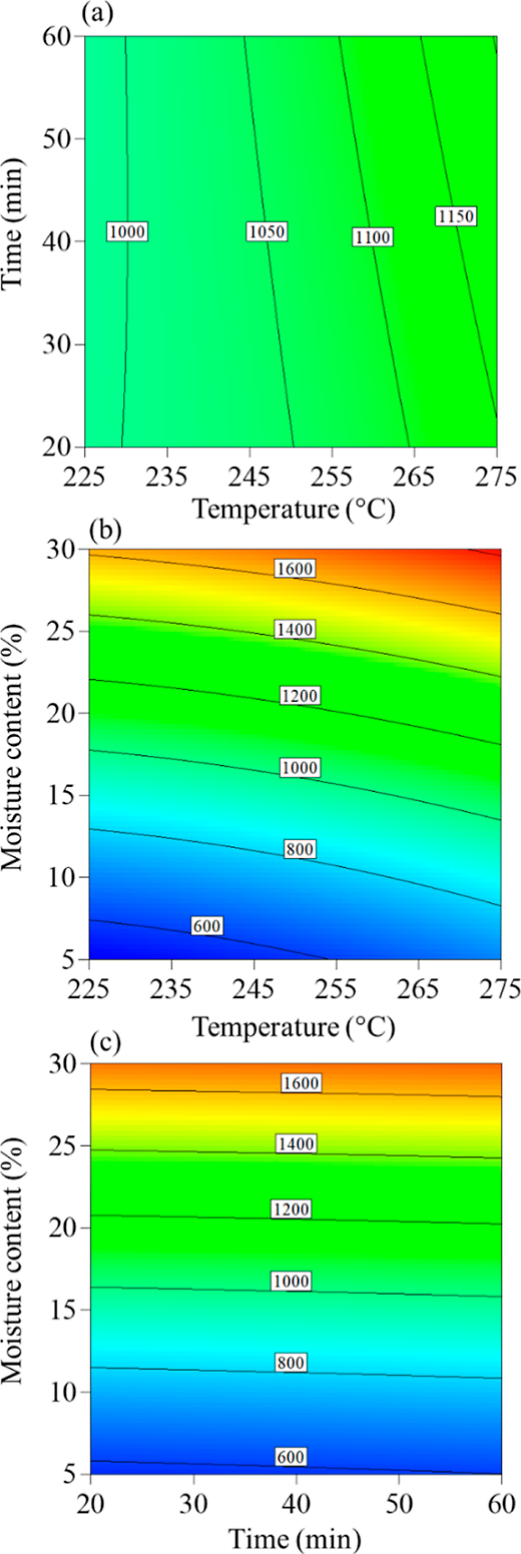
Contour results for *Q*_Torr_ (a) as a function of time and temperature; (b) as a function of moisture and temperature; and (c) as a function of moisture and time.

**Figure 7:**
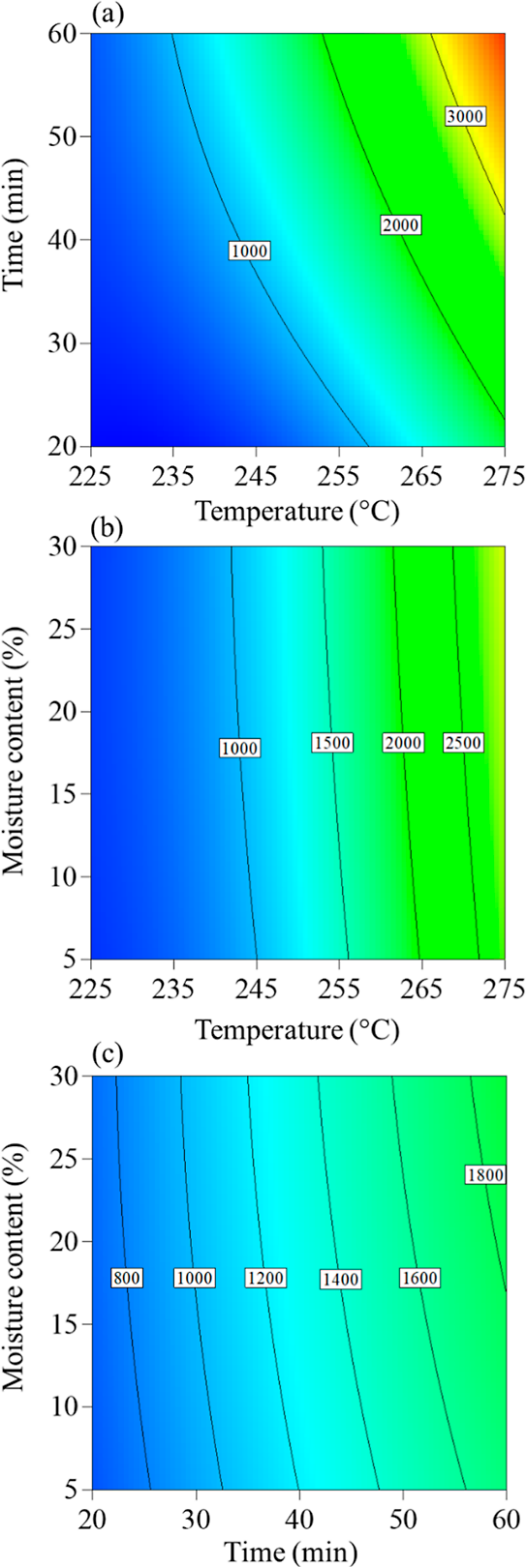
Contour results for irreversibility (a) as a function of time and temperature, (b) as a function of moisture and temperature, and (c) as a function of moisture and time.

This section evaluates the influence of the residence time and the raw biomass moisture level on process performance. Figures 6 and 7 show two-dimensional contour graphics depicting the behavior of consumed heat (*Q*_Torr_) and the irreversibility of the torrefaction process (I), respectively. These graphs cover a torrefaction temperature range of 225–275 °C, a time range of 20–60 min, and raw biomass moisture levels from 5 to 30%.

The heat demand (*Q*_Torr_) varied from 522 to 1853 kJ h^–1^. Figure 6b,c indicates that the initial moisture content significantly influences the *Q*_Torr_. This is due to the additional heat needed to evaporate water, overcome vaporization resistance, and increase the specific heat of water. These data are crucial when considering the possibility of using wet biomass since the drying stage is the main energy consumer, accounting for approximately 76% to 81% of the total consumption, depending on the initial conditions and process inputs.^22,23^ Drying biomass using alternative energy sources before torrefaction, such as sun drying, would considerably increase the process efficiency.

The irreversibility of process (I) ranged from 326 to 3993 kJ·h^–1^, as illustrated in Figure 7a,b, with treatment temperature as the main factor for irreversibility. The irreversibility of the system increased with rising temperature.^46^ Thermal energy has a limited capacity to produce useful work. This difficulty arises because entropy increases over time in isolated systems, making thermal energy transfer on a macroscopic scale inherently irreversible. After conversion into heat and its dispersion, fully recovering this heat and converting it back into useful energy without further increasing the environment’s entropy is impractical.

To avoid wasting useful energy, consider that the use of external sources such as solar energy can reduce losses. The Earth receives exergetic energy from the sun’s radiation as an open system, but a significant portion is radiated back into the universe. By employing solar radiation in drying, it is possible to improve the system’s performance and minimize associated irreversibility.^68^ Moreover, optimizing the torrefaction temperature and residence time and efficiently using byproducts such as bio-oil and torrefaction gas can improve chemical reactions and reduce energy and exergy losses.

## 4 Conclusions

A torrefaction process for UFW was modeled by using Aspen Plus V12.1 software and validated against experimental data, achieving deviations below 5%. The model accurately predicted biomass distribution, energy requirements, and process irreversibilities. Energy consumption ranged from 368 kJ·h^–1^ for light torrefaction to 1853 kJ·h^–1^ for severe torrefaction, while process irreversibilities increased from 326 kJ·h^–1^ to 3993 kJ·h^–1^, corresponding to exergy destruction rates of 3% to 16%. These results highlight the effectiveness of exergetic analysis in identifying energy losses and in guiding process improvements.

Compared to high-temperature pyrolysis, torrefaction offers lower energy consumption and enhanced product stability, making it a competitive biomass pretreatment technology under appropriate conditions. Optimizing the temperature, residence time, and feedstock properties is crucial to achieving efficient and balanced outcomes. For example, higher temperatures improve energy density but reduce the solid yield, emphasizing the need to assess the process further while considering economic and environmental consequences. Future research should investigate torrefaction under alternative atmospheres, such as CO_2_ or flue gas, to further enhance efficiency and product properties.

The validated modeling framework developed in this study provides a robust tool for assessing torrefaction efficiency, supporting its application in commercial-scale renewable energy systems. These findings contribute to advancing sustainable biomass utilization and carbon-neutral energy strategies, laying the groundwork for a broader adoption of torrefaction technology.

## Supporting Information Available

Biomass modeling with correlations for density, calorific value, and heat capacity; torrefaction modeling with a reaction model, kinetic parameters, and volatiles composition; and exergy analysis with the chemical exergy of elements and compounds (PDF)

## Index Summary

A: ashes
*a*: coefficients
C: carbon
Cl: chlorine
*C*_P_: heat capacity
CCD: central composite face-centered design
*d*: dry
dm: dry and mineral matter-free
EF: enhancement factor
EY: energy yield
EMCI: energy-mass-*co*-benefit-index
*E*_x_: exergy
*e*_ch_^0^: standard chemical exergy
FC: fixed carbon
*h*: enthalpy
H: hydrogen
HHV: higher heating value
*I*: irreversibility
IGT: Institute of Gas Technology
LCA: life cycle assessment
MM: mineral matter
N: nitrogen
O: oxygen
*P*: pressure
*R*: universal gas constant
*s*: entropy
S: sulfur
*S*_o_: organic sulfur
*S*_p_: pyritic sulfur
*S*_t_: total
*T*: temperature
UFW: urban forest waste
υ: stoichiometric coefficient
VM: volatile material
*w*: weight fraction
*x*: molar fraction
*Y*: mass yield
ρ: specific density
Δ*g*_r_^0^: standard Gibbs free energy of formation
Δ_c_*h*: specific heat of combustion
Δ_f_*h*: specific heat of formation
