# Supplementary material for: Simulation and Thermodynamic Evaluation of Woody Biomass Waste Torrefaction
Source: ACS Omega. 2025 Jan 21;10(4):3585–97. doi: 10.1021/acsomega.4c08299 (PMC11799986; doi:10.1021/acsomega.4c08299)
Supplement: Supplementary file 1 — ao4c08299_si_001.pdf [file ao4c08299_si_001.pdf]

Simulation and Thermodynamic Analysis of the Torrefaction of Woody Biomass Waste

Thiago da Silva Gonzales [a], Simone Monteiro [a], Giulia C. Lamas [a], Pedro P. O. Rodrigues [a], Mario B. B. Siqueira [a], Luis Alberto Follegatti Romero [b], Edgar A. Silveira [a*]

a. University of Brasília, Mechanical Sciences Graduate Program, Laboratory of Energy and Environment, Brasilia-DF, 70910-900, Brazil.
b. Laboratory of Separation and Purification Engineering (LaSPE), Department of Chemical Engineering (PQI), Polytechnic School (EP), University of São Paulo (USP), SP, São Paulo, Brazil

* Corresponding author E-mail: edgar.silveira@unb.br

2. Methodology

2.1 Biomass feedstock modeling

Table S1. Parameter values for the calculation of the Coal Density and Heat of Combustion Correlations (Boie Correlation).

Parameter name	DCOALIGT DENIGT[a]	HCOALGEN BOIEC[b]
Symbol	Value	Value
a_{1i}	0.4397	151.2
a_{2i}	0.1223	499.77
a_{3i}	-0.01715	45.0
a_{4i}	0.001077	-47.7
a_{5i}	-	27.0
a_{6i}	-	-189.0

Adapted from [1] [a] Coal Density; [b] Heat of Combustion Correlations (Boie Correlation)

Table S2. Parameter values for the calculation of the Heat Capacity Kirov Correlations.

$a_{I,jn}$ j \ n		HCOALGEN CP1C[c] n=1	n=2	n=3	n=4
j=1	Moisture	1.0	0	0	0
j=2	Fixed Carbon	0.165	$6.8x10^{-4}$	$-4.2x10^{-7}$	0
j=3	Primary Volatile Matter	0.395	$8.1x10^{-4}$	0	0
j=4	Secondary Volatile Matter	0.71	$6.1x10^{-4}$	0	0
j=5	Ash	0.18	$1.4x10^{-4}$	0	0

Adapted from [1] [c]Heat Capacity Kirov Correlations. I = Component; j = Contituent

Table S3. Proximate and ultimate compositions of UFW used as input for simulation [2].

**Properties**	**Feedstock**
**Moisture content (%)**	30.00
**Proximate analysis (%)**	
Fixed carbon (FC)	17.90
Volatile matter (VM)	77.61
Ashes	4.49
**Ultimate analysis (%)**	
C	44.91
H	7.25
N	0.64
O [a]	42.71
H/C	1.92
O/C	0.71
**Higher heating value (MJ kg^{-1})**	
HHV	19.79

[a] determined by difference: O = total mass – (C + H + N + ashes)

2.2 Torrefaction plant modeling

In torrefaction, the emphasis is on the transformation of biomass with a controlled release of volatile compounds, resulting in a more stable material with optimized energy properties. Studies on wood torrefaction indicated that mass loss is mainly due to the decomposition of hemicelluloses [3]. Therefore, a two-step (Figure S1) consecutive reaction model was applied. The model is based on the hemicellulose degradation model and uses a

competitive first-order reaction mechanism [4,5]. This model is widely used to describe the torrefaction kinetics of various biomasses, enabling the decomposition of biomass into its pseudo-components. It illustrates the temporal evolution of mass loss during treatment and has been employed in numerous studies for modeling biomass decomposition [6–11].

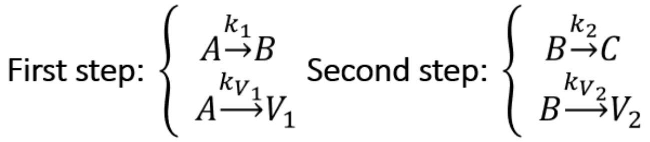

Figure S1. Diagram of the two-step reaction model. Adapted from [2]

According to the model, A represents the original biomass undergoing pre-treatment, B is an intermediate solid product, C is the residual solid product. The volatile fraction released during the process is ascribed to V_1 and V_2. Those reactions follow the first-order model [3,4,12,13], resulting in Eqs. (1–5) by integration.

$$Y_A = e^{[-(k_1+k_{V_1})t]} \tag{1}$$

$$Y_B = \frac{k_1}{(k_2+k_{V_2})-(k_1+k_{V_1})}\left[e^{[-(k_1+k_{V_1})t]} - e^{[-(k_2+k_{V_2})t]}\right] \tag{2}$$

$$Y_{V_1} = \frac{k_{V_1}}{(k_1+k_{V_1})}\left[1 - e^{[-(k_1+k_{V_1})t]}\right] \tag{3}$$

$$Y_C = \frac{k_1 k_2}{(k_2+k_{V_2})-(k_1+k_{V_1})}\left[\frac{e^{[-(k_2+k_{V_2})t]}}{(k_2+k_{V_2})} - \frac{e^{[-(k_1+k_{V_1})t]}}{(k_1+k_{V_1})}\right] + \frac{k_1 k_2}{(k_2+k_{V_2})(k_1+k_{V_1})} \tag{4}$$

$$Y_{V_2} = \frac{k_1 k_{V_2}}{(k_2+k_{V_2})-(k_1+k_{V_1})}\left[\frac{e^{[-(k_2+k_{V_2})t]}}{(k_2+k_{V_2})} - \frac{e^{[-(k_1+k_{V_1})t]}}{(k_1+k_{V_1})}\right] + \frac{k_1 k_{V_2}}{(k_2+k_{V_2})(k_1+k_{V_1})} \tag{5}$$

The terms k_1, k_{V_1}, k_2 and k_{V_2} denote the four Arrhenius kinetic parameters (Table S4) and can be determined by fitting the experimental thermogravimetric (TG) curves (mass loss) and numerical predicted data. Equation (6) (Arrhenius equation) describes the relationship between pre-exponential factor (A_{0_i}), activation energy (E_{a_i}), universal gas constant, denoted as R = 8.314 J K^{-1} mol^{-}, and temperature T (in K). The kinetic parameters were determined in previous studies [2].

$$k_i = A_{0_i} e^{\left(\frac{-Ea_i}{RT}\right)} \tag{6}$$

Table S4. Kinetic parameters.

i	A_{0_i} (s^{-1})	Ea_i ($J.mol^{-1}$)
1	2.78×10^9	1.25×10^5
V_1	1.46×10^7	1.13×10^5
2	5.35×10^0	5.03×10^4
V_2	6.08×10^5	1.04×10^5

Adapted from [2].

At the end of the process, the total final composition of the mass fraction of the solids (Y_{TS}) is determined by the sum of masses Y_A, Y_B, and Y_C, while total final composition of the mass fraction of the volatiles (Y_{TV}) is composed of the sum of V_1 and V_2 [8]. Y_{TV} can be subdivided according to the proportion of each of the nine components present in V_1 and V_2.

Bates and Ghoniem [12] studied the modeling and evolution of volatile products from the torrefaction of willow, where the composition of the volatiles released during torrefaction was qualitatively and quantitatively analyzed. These values served as the basis for the composition of the volatiles of the present work (Table S5). In V_1, highly oxygenated species such as water, carbon dioxide, and acetic acid are found, while V_2 is mainly composed of

condensable volatiles such as lactic acid, methanol, acetic acid, water, formic acid, and hydroxy acetone [12].

Table S5. Composition of volatiles

**Component**	**Volatiles**	
	$\boldsymbol{V_1}$	$\boldsymbol{V_2}$
Acetic Acid (CH_3COOH)	14.8	16.1
Water (H_2O)	48.1	7.6
Formic Acid (HCOOH)	5.3	5.1
Methanol (CH_3OH)	4.2	30.1
Lactic Acid (CH_3-CH(OH)-COOH)	1.3	31.3
Furfural (C_4H_3OCHO)	1.1	0.0
Hydroxyacetone (CH_3COCH_2OH)	0.6	9.7
Carbon Dioxide (CO_2)	20.4	0.0
Carbon Monoxide (CO)	4.2	0.1

Adapted from [14].

A previous study provided the linear correlation between torrefaction temperature and proximate properties (equations with R^2 of ~0.98). Those linear correlations were applied within the Aspen Plus® modeling to calculate the FC (Eq. (7)), VM (Eq. (8)) and Ash (Eq. (9)) of the torrefied solid product [2].

$$FC_{ST} = -56.633Y_{ST} + 73.724 \quad (7)$$

$$VM_{ST} = 61.629Y_{ST} + 16.905 \quad (8)$$

$$Ash_{ST} = 100 - FC_{ST} - VM_{ST} \quad (9)$$

2.2.2 Exergetic Analysis

Table S6. Standard chemical exergy

Element	Value used ex_q^0 [kJ.mol^{-1}]	Calculated	Literature
Water (l)	0.90[a]	-	0.90[a]
Nitrogen	0.72[a]	-	0.72[a]
Acetic Acid	920.30	920.30	919.0[b]
Water (g)	9.50[a]	-	9.50[a]
Formic Acid	298.00	298.00	301.3[b]
Methanol (g)	722.30[a]	-	722.30[a]
Lactic Acid	1444.20	1444.20	1545.78[c]
Furfural	2422.20	2422.20	2407.54[d]
Hydroxyacetone	1655.40	1655.40	-
Carbon Dioxide	19.87[a]	-	19.87[a]
Carbon Monoxide	275.10[a]	-	275.10[a]

[a][15]; [b][16]; [c][17]; [d][18].

3. Results

Table S7. Stream data from Aspen Plus for torrefaction validation for light, mild, and severe torrefaction cases (60-minute reaction time and P = 1 atm), detailing flow rates, compositions, and temperatures of each component.

**Aspen Plus Blocks**		**Dry Biomass**			**N2 (Cold)**			$Separ_{out}$			$Biomass_{torr}$		
**Temperature**	**°C**	**25**	**25**	**25**	**25**	**25**	**25**	**225**	**250**	**275**	**225**	**250**	**275**
**Mass Flows**	**kg.h^{-1}**	**1.000**	**1.000**	**1.000**	**0.001**	**0.001**	**0.001**	**0.059**	**0.130**	**0.248**	**0.942**	**0.871**	**0.753**
**Biomass**	**kg.h^{-1}**	1.000	1.000	1.000	-	-	-	0.000	0.000	0.000	-	-	-
**TorrBiomass**	**kg.h^{-1}**	-	-	-	-	-	-	0.000	0.000	0.000	0.942	0.871	0.753
**Nitrogen**	**kg.h^{-1}**	-	-	-	0.001	0.001	0.001	0.001	0.001	0.001	-	-	-
**Acetic Acid**	**kg.h^{-1}**	-	-	-	-	-	-	0.009	0.020	0.039	-	-	-
**Water**	**kg.h^{-1}**	-	-	-	-	-	-	0.025	0.040	0.046	-	-	-
**Formic Acid**	**kg.h^{-1}**	-	-	-	-	-	-	0.003	0.007	0.013	-	-	-
**Methanol**	**kg.h^{-1}**	-	-	-	-	-	-	0.004	0.020	0.057	-	-	-
**Lactic Acid**	**kg.h^{-1}**	-	-	-	-	-	-	0.003	0.018	0.057	-	-	-
**Furfural**	**kg.h^{-1}**	-	-	-	-	-	-	0.001	0.001	0.001	-	-	-
**Hydroxyacetone**	**kg.h^{-1}**	-	-	-	-	-	-	0.001	0.006	0.018	-	-	-
**Carbon Dioxide**	**kg.h^{-1}**	-	-	-	-	-	-	0.010	0.015	0.014	-	-	-
**Carbon Monoxide**	**kg.h^{-1}**	-	-	-	-	-	-	0.002	0.003	0.003	-	-	-
**Biomass and biocoal properties**													
**PROXANAL**													
**Moisture**	**%**	0.00	0.00	0	-	-	-	-	-	-	0.00	0.00	0.00
**FC**	**%**	17.9	17.9	17.9	-	-	-	-	-	-	20.37	24.40	31.10
**VM**	**%**	77.61	77.61	77.61	-	-	-	-	-	-	74.97	70.58	63.29
**Ash**	**%**	4.49	4.49	4.49	-	-	-	-	-	-	4.66	5.02	5.61
**ULTANAL**													
**Carbon**	**%**	44.91	44.91	44.91	-	-	-	-	-	-	46.42	47.77	49.41
**Hydrogen**	**%**	7.25	7.25	7.25	-	-	-	-	-	-	7.23	7.14	6.87
**Nitrogen**	**%**	0.64	0.64	0.64	-	-	-	-	-	-	0.68	0.73	0.85
**Oxygen**	**%**	42.71	42.71	42.71	-	-	-	-	-	-	40.90	39.19	36.91
**Energy flow**													
**Qtorr**	**kJ.h^{-1}**										368	441	561

References

[1] Aspen Technology. Aspen Plus Help. V12.1. Cambridge, MA: Aspen Technology, Inc.; 2021.

[2] Silveira EA, Santanna MS, Barbosa Souto NP, Lamas GC, Galvão LGO, Luz SM, et al. Urban lignocellulosic waste as biofuel: thermal improvement and torrefaction kinetics. J Therm Anal Calorim 2023. https://doi.org/10.1007/s10973-022-11515-0.

[3] Prins MJ, Ptasinski KJ, Janssen FJJG. Torrefaction of wood: Part 1. Weight loss kinetics. J Anal Appl Pyrolysis 2006;77:28–34. https://doi.org/10.1016/J.JAAP.2006.01.002.

[4] Di Blasi C, Lanzetta M. Intrinsic kinetics of isothermal xylan degradation in inert atmosphere. J Anal Appl Pyrolysis 1997;40–41:287–303. https://doi.org/10.1016/S0165-2370(97)00028-4.

[5] Lanzetta M, Di Blasi C. Pyrolysis kinetics of wheat and corn straw. J Anal Appl Pyrolysis 1998;44:181–92. https://doi.org/10.1016/S0165-2370(97)00079-X.

[6] Bach QV, Skreiberg Ø, Lee CJ. Process modeling and optimization for torrefaction of forest residues. Energy 2017;138:348–54. https://doi.org/10.1016/j.energy.2017.07.040.

[7] Bach QV, Skreiberg Ø, Lee CJ. Process modeling for torrefaction of birch branches. Energy Procedia 2017;142:395–400. https://doi.org/10.1016/J.EGYPRO.2017.12.062.

[8] Haseli Y. Process Modeling of a Biomass Torrefaction Plant. Energy and Fuels 2018;32:5611–22. https://doi.org/10.1021/acs.energyfuels.7b03956.

[9] Manouchehrinejad M, Mani S. Process simulation of an integrated biomass torrefaction and pelletization (iBTP) plant to produce solid biofuels. Energy Conversion and Management: X 2019;1. https://doi.org/10.1016/j.ecmx.2019.100008.

[10] Mukherjee A, Okolie JA, Niu C, Dalai AK. Experimental and Modeling Studies of Torrefaction of Spent Coffee Grounds and Coffee Husk: Effects on Surface Chemistry and Carbon Dioxide Capture Performance. ACS Omega 2022;7:638–53. https://doi.org/10.1021/acsomega.1c05270.

[11] Onsree T, Jaroenkhasemmeesuk C, Tippayawong N. Techno-economic assessment of a biomass torrefaction plant for pelletized agro-residues with flue gas as a main heat source. Energy Reports 2020;6:92–6. https://doi.org/10.1016/j.egyr.2020.10.043.

[12] Bates RB, Ghoniem AF. Biomass torrefaction: Modeling of volatile and solid product evolution kinetics. Bioresour Technol 2012;124:460–9. https://doi.org/10.1016/j.biortech.2012.07.018.

[13] Prins MJ, Ptasinski KJ, Janssen FJJG. Torrefaction of wood: Part 2. Analysis of products. J Anal Appl Pyrolysis 2006;77:35–40. https://doi.org/10.1016/J.JAAP.2006.01.001.

[14] Bates RB, Ghoniem AF. Biomass torrefaction: Modeling of volatile and solid product evolution kinetics. Bioresour Technol 2012;124:460–9. https://doi.org/10.1016/j.biortech.2012.07.018.

[15] Moran MiJ, Shapiro HN, Boettner DD, Bailey MB. Princípios de Termodinâmica para Engenharia. 8th ed. Rio de Janeiro - RJ: LTC — Livros Técnicos e Científicos Editora Ltda; 2018.

[16] Morris DR, Szargut J. Standard chemical exergy of some elements and compounds on the planet earth. Energy 1986;11:733–55. https://doi.org/10.1016/0360-5442(86)90013-7.

[17] Aghbashlo M, Mandegari M, Tabatabaei M, Farzad S, Mojarab Soufiyan M, Görgens JF. Exergy analysis of a lignocellulosic-based biorefinery annexed to a sugarcane mill for simultaneous lactic acid and electricity production. Energy 2018;149:623–38. https://doi.org/10.1016/j.energy.2018.02.063.

[18] Wiranarongkorn K, Im-orb K, Panpranot J, Maréchal F, Arpornwichanop A. Exergy and exergoeconomic analyses of sustainable furfural production via reactive distillation. Energy 2021;226. https://doi.org/10.1016/j.energy.2021.120339.
